# COVID-19 mRNA Vaccines as Antigen-Agnostic Immunomodulators: A Repurposing Perspective

**DOI:** 10.3390/medsci14050572

**Published:** 2026-09-15

**Authors:** Piercarlo Minoretti, Enzo Emanuele

**Affiliations:** 1 Department of Social Sciences, Miguel de Cervantes European University (UEMC), 47012 Valladolid, Spain; scientific.direction@studiominoretti.it; 2 Studio Minoretti, I-23848 Oggiono, LC, Italy; 3 2E Science, I-27038 Robbio, PV, Italy

**Keywords:** COVID-19 mRNA vaccines, drug repurposing, antigen-agnosticimmunomodulation, type I interferon, immune checkpoint inhibitors, trained immunity, target trial emulation

## Abstract

Since 2020, billions of doses of COVID-19 messenger RNA (mRNA) lipid nanoparticle vaccines have been administered worldwide, generating one of the largest pharmacovigilance databases for a modern therapeutic class. Intriguingly, their immunopharmacologic profile—including the engagement of innate nucleic acid sensors, systemic type I interferon signaling, dendritic cell-mediated cross-priming of CD8+ T cells, and upregulation of programmed cell death ligand 1 (PD-L1)—may extend beyond COVID-19 prophylaxis. Retrospective evidence from six independent cohorts, one of which is a multinational health-data network, indicated that vaccination within 100 days of immune checkpoint inhibitor (ICI) initiation may improve survival of patients with malignancies, including in immunologically cold tumors, although a substantial fraction of the original effect may be attributed to pandemic-era selection bias. Here, we examine the mechanistic basis and available evidence for the repurposing of licensed COVID-19 mRNA vaccines as antigen-agnostic immunomodulators. We identified five candidate contexts for prospective evaluation—namely, (1) PD-L1-negative non-small cell lung cancer initiating ICI therapy, (2) perioperative immune dysfunction in major cardiac surgery, (3) chronic hepatitis B, (4) chronic HIV with persistent immune exhaustion, and (5) sepsis-induced immunoparalysis. Methodological foundations for confirmatory assessment—comprising target trial emulation, Mendelian randomization, and pragmatic trial design—are now sufficiently developed to enable implementation. Prospective evaluation of the antigen-agnostic repurposing hypothesis is operationally feasible, albeit constrained by residual confounding and uncertainty about the durability of vaccine-induced innate reprogramming.

## 1. Introduction

The clinical deployment of mRNA-lipid nanoparticle (LNP) vaccines against SARS-CoV-2 has generated the largest pharmacovigilance database ever compiled, with billions of doses administered worldwide across the BNT162b2 (Pfizer-BioNTech) and mRNA-1273 (Moderna) platforms and follow-up extending across four years and multiple continents [1]. The technical innovations of these vaccines rest on nucleoside modification of mRNA—which evades innate immune detection while preserving protein translation [2]—and on LNP formulation—which protects synthetic mRNA and delivers it to antigen-presenting cells in tissue and draining lymph node compartments [3]. Notably, both components are characterized by measurable immunomodulatory effects independent of the antigen they convey—including activation of innate sensors, induction of type I interferon (IFN-I), and reprogramming of myeloid cells [4,5]. In addition, LNPs have been described as intrinsic adjuvants even when administered without antigenic cargo [6]—a property that has shaped the broader trajectory of vaccinology over the past decade [7].

Drug repurposing has historically produced some of the most consequential therapeutic advances when an existing licensed product can be redirected against a new indication without retracing the full sequence of safety and pharmacokinetic development [8]. Licensed COVID-19 mRNA vaccines satisfy these criteria—combining industrial-scale production, population-level safety data [1,9], and marginal cost relative to other immunomodulators. A recent observation has placed this potential under direct scrutiny. Receipt of a COVID-19 mRNA vaccine within 100 days of immune checkpoint inhibitor (ICI) initiation has been associated with significant gains in median and three-year overall survival across multiple retrospective oncologic cohorts—including in patients with immunologically cold tumors—and was supported by preclinical evidence of IFN-I induction, CD8+ T cell cross-priming, and tumor PD-L1 upregulation [5]. These results have prompted discussion of the possibility that the repurposing of COVID-19 mRNA vaccines may serve as a translatable approach to potentiate the efficacy of ICI-based cancer immunotherapy [10]. In parallel, a distinct line of investigation has examined a mechanistically separate repurposing strategy in which intratumoral administration of BNT162b2 in previously immunized mice can redirect pre-existing anti-SARS-CoV-2 memory immunity toward spike-expressing tumor cells, reshape the tumor microenvironment, and induce antigen spreading to endogenous tumor epitopes—with anti-PD-L1 therapies further amplifying these effects in immunologically cold models [11]. Although the strongest evidence to date is in the field of oncology, the molecular cascade engaged by the systemic intramuscular route operates upstream of any tumor compartment and converges on innate sensor activation, IFN-I induction, and myeloid reprogramming, all of which are also implicated in non-tumoral conditions where modulation of host immunity can be therapeutically valuable.

In this narrative review, we sought to assess the mechanistic rationale and clinical evidence underlying the repurposing of licensed COVID-19 mRNA vaccines as antigen-agnostic immunomodulators, to delineate the methodological conditions necessary for prospective testing of the retrospective signal, and to identify the clinical contexts in which a definitive trial would be most informative.

The evidence assembled here was identified through a narrative rather than a systematic search strategy, a choice dictated by the framework-integrative scope of the argument, which spans immunopharmacology, oncology, infectious disease and critical care and therefore cannot be reduced to a single question structured around population, intervention, comparator and outcome. PubMed, Scopus, and ScienceDirect were searched from 1 January 2020 to 31 August 2026 using combinations of terms covering the platform (COVID-19 mRNA vaccine, BNT162b2, mRNA-1273, lipid nanoparticle), the proposed mechanism (type I interferon, trained immunity, cross-priming, PD-L1, antigen-agnostic immunomodulation) and each candidate setting (immune checkpoint inhibitor, cardiac surgery, chronic hepatitis B, HIV reservoir, sepsis-induced immunoparalysis). Retrieval was restricted to reports published in English, and the reference lists of retrieved articles were screened for studies not captured by the database search, through which twenty-eight foundational reports predating the search window were also identified. Where both were available for a given claim, primary studies, randomized trials and large cohorts were preferred over narrative sources, and priority was given to work published from 2021 onward, which accounts for 103 of the 134 references cited, whereas earlier literature was retained where it establishes a mechanism or a clinical precedent that subsequent work assumes.

## 2. Mechanistic Foundation: From LNP Delivery to Checkpoint-Relevant Immune Activation

### 2.1. Lipid Nanoparticle Delivery and Innate Sensing of mRNA Cargo

**Figure 1:**
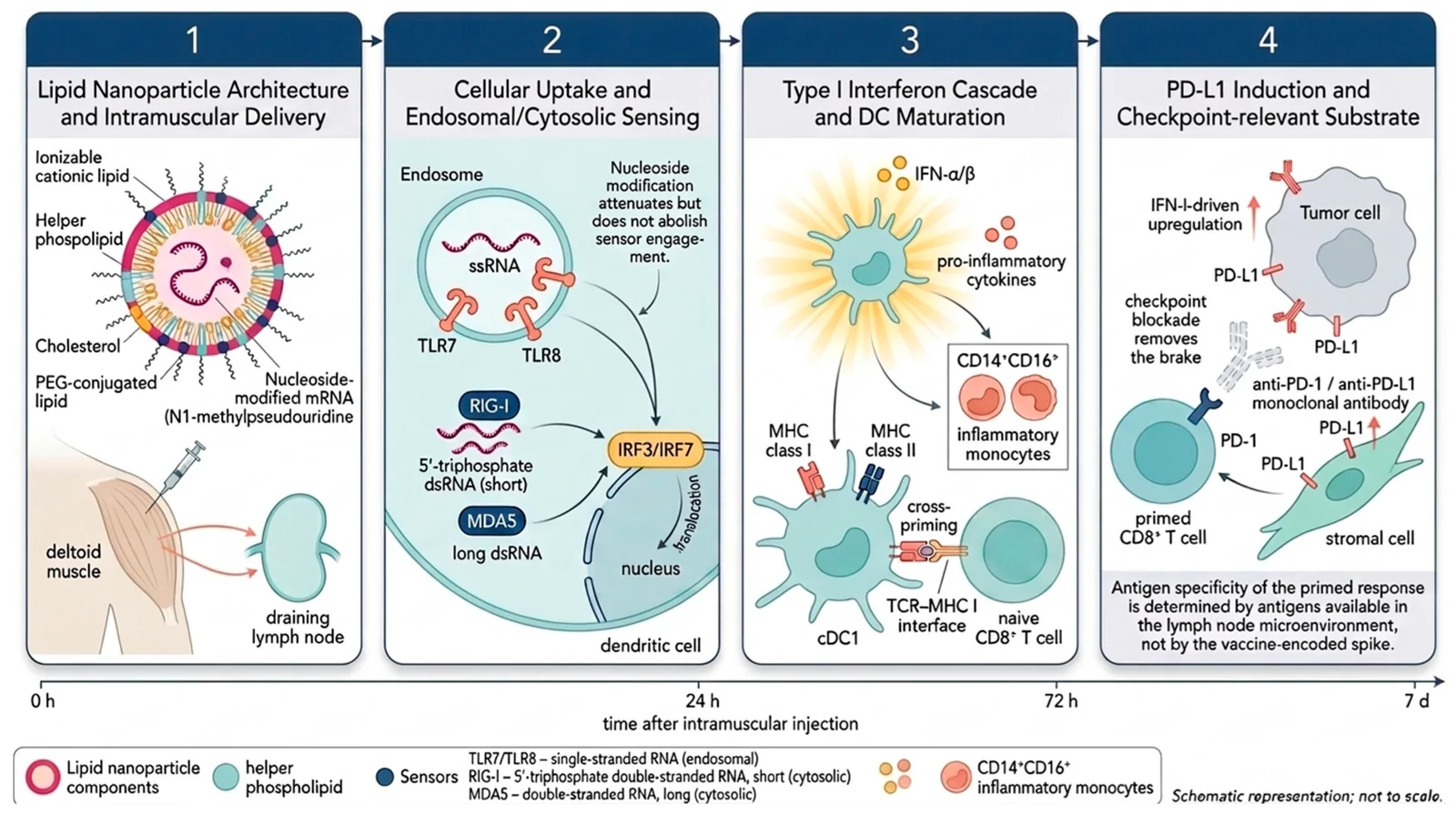
**Antigen-agnostic immunomodulation by licensed COVID-19 mRNA lipid nanoparticle vaccines.** Molecular and cellular cascade through which licensed mRNA lipid nanoparticle (LNP) vaccines act as antigen-agnostic immunomodulators. The lipid nanoparticle carrier, composed of an ionizable cationic lipid, a helper phospholipid, cholesterol, and a polyethylene glycol (PEG)-conjugated lipid, delivers nucleoside-modified messenger RNA to dendritic cells and macrophages. Intracellular sensing of the modified mRNA cargo by Toll-like receptors 7 and 8 within the endosomal compartment, together with cytosolic sensing of RNA species by retinoic acid-inducible gene I (RIG-I), which detects short 5′-triphosphate double-stranded RNA, and melanoma differentiation-associated protein 5 (MDA5), which detects long double-stranded RNA, triggers a coordinated type I interferon (IFN-I) cascade. IFN-I signaling drives dendritic cell maturation, upregulation of major histocompatibility complex (MHC) class I and II, and conventional type 1 dendritic cell (cDC1)-mediated cross-priming of CD8+ T cells against locally available antigens, including tumor-associated antigens in patients with malignancy. The same IFN-I cascade induces programmed cell death ligand 1 (PD-L1) expression on tumor and stromal cells, providing the molecular substrate on which immune checkpoint inhibitor therapy operates. The cascade is independent of the spike antigen specificity encoded by the vaccine and constitutes the mechanistic foundation for the antigen-agnostic repurposing hypothesis. Abbreviations: cDC1, conventional type 1 dendritic cell; IFN-I, type I interferon; LNP, lipid nanoparticle; MDA5, melanoma differentiation-associated protein 5; MHC, major histocompatibility complex; mRNA, messenger RNA; PD-L1, programmed cell death ligand 1; PEG, polyethylene glycol; RIG-I, retinoic acid-inducible gene I; TLR, Toll-like receptor. *Image generated with FigureLabs.ai Scientific Illustration Software (web-based application; accessed on August 2026).*

The immunomodulatory effects underlying the repurposing hypothesis arise from the molecular architecture of the mRNA-LNP platform (Figure 1) [12]. The lipid nanoparticle carrier—an ionizable cationic lipid, a helper phospholipid, cholesterol, and a polyethylene glycol (PEG)-conjugated lipid [13]—functions not only as a delivery system but also as an intrinsic adjuvant [3], a dual property that underlies the antigen-agnostic immunostimulatory potential exploited in oncologic settings.

The pharmacokinetic determinant most relevant to oncologic adjuvant activity is the delivery of LNP cargo to the draining lymph node. After intramuscular administration, the particle is taken up by resident antigen-presenting cells (APCs) and carried by interstitial flow to the draining lymph node, where it engages a second wave of APCs [3]. The same biodistribution had been documented in systemically delivered RNA-LNP formulations designed for splenic targeting, in which strong IFN-α release and dendritic cell maturation were induced in lymphoid organs [14]. This anatomical compartment is also a key site of immune checkpoint blockade activity, because PD-L1 expressed by dendritic cells in tumor-draining lymph nodes (TDLNs) can limit the priming of tumor-specific T cells [15].

This spatial overlap is the structural basis for the proposed synergy. The ionizable lipid component drives local inflammatory signaling, recruits neutrophils and monocytes, and activates innate sensing pathways in APCs independently of the cargo [3]. Accordingly, the immunostimulatory activity of COVID-19 mRNA vaccines in the repurposing setting depends on the platform rather than on the encoded spike protein, a view consistent with the cancer mRNA vaccine literature, which regards innate sensing of the mRNA cargo together with the LNP carrier as determinants of antitumor immunity [16].

The molecular sensors engaged by the mRNA cargo govern the quality and intensity of the downstream interferon response. Endosomal Toll-like receptors 7 and 8 (TLR7 and TLR8) detect single-stranded RNA, whereas residual double-stranded RNA contaminants and structured RNA species activate the cytosolic receptors retinoic acid-inducible gene I (RIG-I) and melanoma differentiation-associated protein 5 (MDA5) [3,17]. Cyclic GMP-AMP synthase and the stimulator of interferon genes pathway may further contribute in response to cellular stress and the release of DNA from damaged APCs. Nucleoside modification, originally introduced to mitigate innate sensing of unmodified RNA through TLR-mediated pathways [2], attenuates this signaling only in part; the pseudouridine substitution that yields a minimally immunogenic mRNA platform with high translational efficiency [18] preserves, in BNT162b2 and mRNA-1273, sufficient residual TLR7/8 and RIG-I/MDA5 activity to induce a measurable systemic IFN-I response in healthy adults [5], which in the repurposing context is likely to underlie the antigen-agnostic immunomodulatory effects observed in oncologic cohorts.

The temporal dynamics of innate activation are equally relevant to prospective trial design. After a single intramuscular dose the local innate response peaks within 24 h and subsides over the following days, with transient elevations in serum cytokines and chemokines that recruit additional myeloid cells to the draining lymph node [3,4]. Following a second dose the response increases in both magnitude and quality, a pattern attributed to vaccine-induced reprogramming of the innate compartment analogous to trained immunity [19]. Consistent with this, an additional dose of BNT162b2 restored humoral and cellular responses in patients with non-small cell lung cancer (NSCLC) receiving ICI therapy [20]. Prospective oncologic studies should therefore evaluate boosted regimens rather than limiting assessment to the primary series.

### 2.2. Type I Interferon Cascade and Antigen-Agnostic Cross-Priming

Systems-level profiling of BNT162b2 recipients revealed a substantial increase in plasma IFN-γ and a transcriptional program indicative of innate antiviral activation after the second dose [4], with direct measurement of circulating IFN-I subsequently confirmed in healthy volunteers [5]. In the same study, the frequency of a myeloid cluster enriched in interferon-response transcription factors and depleted of AP-1 transcription factors increased by approximately two orders of magnitude, together with expansion of CD14+CD16+ inflammatory monocytes, evidence that in humans the platform reconfigures innate immunity in ways that favor adaptive priming, consistent with the retrospective oncologic signal [5]. In parallel, spatiotemporal studies of germinal center kinetics have shown that mRNA vaccination sustains germinal center activity in the draining lymph node with prolonged antigen retention and affinity maturation [21], providing an interface at which tumor-draining APCs may engage vaccine-conditioned T follicular helper cells.

The mechanistic link between IFN-I and antitumor CD8+ T-cell priming centers on conventional type 1 dendritic cells (cDC1). IFN-I induces cDC1 maturation, migration to the draining lymph node, and cross-presentation of exogenous antigens on MHC class I [22], the pathway required for activating naïve CD8+ T cells against antigens not expressed by the APC itself [23]; this explains how a vaccine-induced innate program can promote priming against tumor antigens unrelated to the encoded cargo [17]. When tumor-derived material reaches the lymph node through afferent lymphatic drainage, IFN-I-activated cDC1 capture, process and present it to naïve T cells, in a spatial arrangement made more precise by the ipsilateral homing of recall B cell responses and by subcapsular sinus macrophage-directed antigen capture [24].

Once the innate cascade is operating in the draining lymph node, the antigen specificity of the cross-primed T cell response is determined by the antigens available in that microenvironment rather than by the antigen encoded by the vaccine [5]. Direct experimental support derives from preclinical work with RNA-lipid particle aggregates engineered to maximize RIG-I activation in stromal cells, which drove T cell trafficking and tumor rejection when the encoded mRNA was not tumor-derived, although in that model the survival benefit was greater with tumor-specific than with control mRNA [17]. For licensed COVID-19 mRNA vaccines, whose spike protein is not a tumor antigen, the LNP and modified mRNA components engage innate sensors and drive IFN-I and DC activation in patterns broadly similar to those produced by personalized tumor mRNA vaccines [23]; repeated SARS-CoV-2 antigen exposure further shapes the polarization of spike-specific T cells, with heightened activation of effector memory CD8+ T cells in vaccinated individuals [25].

The mechanism proposed here has an explicit antecedent in the literature on epitope spreading. Determinant spreading was first described in experimental autoimmune encephalomyelitis, where a response initiated against a single determinant diversified to additional intramolecular and intermolecular epitopes under conditions of sustained inflammation and tissue injury [26], and the phenomenon was subsequently generalized across immune-mediated diseases and deliberately exploited in immunotherapy [27]. The antigen-agnostic hypothesis shares that architecture while differing in one respect that governs its therapeutic logic. Innate activation supplies the context in which priming occurs, comprising dendritic cell maturation, upregulation of MHC class I, and costimulation, whereas the specificity of the resulting adaptive response is set by the antigens available within the tissue in which that activation takes place. Two components of the formulation carry this context-setting activity. The ionizable lipid of the LNP behaves as an intrinsic adjuvant independently of any encoded cargo [6], and the nucleoside-modified mRNA retains residual agonism at TLR7/8 and at the cytosolic RNA sensors despite N1-methylpseudouridine substitution [2,18]. Neither component confers antigen specificity, and the encoded spike protein confers a specificity that is irrelevant to the tumor. Accordingly, the innate stimulus alone is not sufficient to generate clinically meaningful antigen-specific immunity in the absence of an available antigen source, and the hypothesis predicts benefit only where a locally accessible antigenic repertoire already exists, such as a tumor bearing neoantigens, a persistently infected hepatocyte compartment, or a residual viral reservoir. This constraint accounts for the dependence of the effect on neoantigen burden and HLA presentation [28], and it bounds the generalizability of the approach across patients, infections, and tumor types.

By contrast, differences between the two licensed mRNA vaccines carry potential implications for any prospective repurposing trial. BNT162b2 and mRNA-1273 share the prefusion-stabilized spike encoded, the N1-methylpseudouridine substitution, and an LNP architecture of comparable design [13], but differ in the ionizable lipid, in the mRNA dose per injection, and in the lipid-to-RNA mass ratio, with the higher-dose SM-102 formulation associated with a more pronounced innate signature in observational comparisons. Because the retrospective oncologic cohorts pooled both vaccines without stratification [5] and the systematic review of vaccine response in ICI-treated patients combined the two products [9], stratified randomization or pre-specified subgroup analysis will be required in any pragmatic trial.

### 2.3. PD-L1 Induction and the Molecular Basis for ICI Synergy

The synergy between mRNA-LNP vaccination and ICI blockade rests on coordinated upregulation of PD-L1 in response to vaccine-induced IFN-I [5]. PD-L1 is a canonical IFN-stimulated gene induced on tumor, myeloid and dendritic cells through interferon receptor signaling in the tumor microenvironment [29], and vaccine-induced IFN-I acts as a physiologic inducer of PD-L1 in the same anatomic compartments where ICI exerts its therapeutic effect [5,15]. In murine models of immunologically cold tumors, mRNA-LNP vaccination produced measurable PD-L1 upregulation on tumor and stromal cells [5], generating the very vulnerability that programmed cell death protein 1 (PD-1) and PD-L1 antibodies are designed to exploit.

PD-L1 induction constrains the vaccine-induced response and, at the same time, creates the therapeutic opportunity. Without concurrent ICI, the IFN-I cascade that drives dendritic cell maturation and cross-priming also recruits inhibitory PD-1/PD-L1 interactions that dampen the newly primed CD8+ T cells [30], whereas an anti-PD-1 or anti-PD-L1 antibody relieves this inhibition [31], converting a self-limiting innate stimulus into a sustained adaptive response, as confirmed preclinically by the requirement for concurrent ICI to achieve maximal efficacy in cold tumor models [5]. Neoantigen burden and HLA presentation determine which clones expand under combined therapy [28], and the compartmentalization of PD-L1 expression in the TDLN [15] locates the therapeutic action where ICI is most active.

## 3. The Cancer Immunotherapy Case

The clearest test of the antigen-agnostic repurposing hypothesis has emerged from oncology, where ICIs have produced durable responses in a subset of patients while leaving most tumors unresponsive [32]. The therapeutic class has since expanded across tumor types, with anti-PD-1 antibody combined with platinum doublets now the standard of care in advanced non-squamous [33] and squamous NSCLC [34], and neoadjuvant ICI-based regimens standard for resectable lung cancer [35] and stage III melanoma [36], with ten-year overall survival data from advanced melanoma confirming the durability of combination immunotherapy [37]. Primary and acquired resistance nonetheless remain the principal obstacles to broader benefit, particularly in immunologically cold tumors with low T cell infiltration and low PD-L1 expression [38]. Two converging observations have placed the repurposing question on the agenda. Systemic administration of LNP-encapsulated, nucleoside-modified mRNA engages innate immune sensors and induces coordinated dendritic cell and lymphocyte activation, a mechanism that overlaps with the principle underlying therapeutic cancer vaccines [23]; and a cluster of six independent retrospective oncologic cohorts, comprising the original MD Anderson analysis and five subsequent replications at cancer centers and national registries across four countries and one multinational health-data network, has associated peri-ICI COVID-19 mRNA vaccination with measurable gains in overall survival, including in PD-L1-negative subgroups [5,39,40,41,42,43].

### 3.1. Clinical Signal and Mechanistic Backbone

COVID-19 mRNA vaccines sensitize tumors to ICI blockade in murine models and across retrospective patient cohorts [5], providing the most articulated case to date for their repositioning as antigen-agnostic adjuvants. In preclinical models across solid tumor types, intramuscular mRNA-LNP vaccines encoding the SARS-CoV-2 spike protein produced a systemic increase in IFN-I signaling consistent with residual engagement of innate nucleic acid sensors at the injection site and in draining lymphoid tissue [2]. The IFN-I surge enabled innate immune cells to cross-prime CD8+ T cells against tumor-associated antigens [5], a mechanism long recognized as a determinant of durable anti-tumor immunity [22]. Concomitant PD-1 or PD-L1 blockade was required for maximal efficacy in cold tumors, which responded by upregulating PD-L1 on tumor and stromal cells, thereby generating the vulnerability that ICIs are designed to exploit [15].

The clinical correlate followed the preclinical mechanism with notable concordance. In healthy human volunteers, BNT162b2 or mRNA-1273 produced measurable rises in circulating IFN-I and in myeloid and lymphoid activation, confirming the antigen-agnostic component of the innate signature elicited by mRNA-LNP platforms [5]. In patients receiving ICI for advanced disease, vaccination within 100 days of ICI initiation was associated with improved median and three-year overall survival across multiple large retrospective cohorts, with adjusted hazard ratios (HRs) of approximately 0.51 (95% confidence interval [CI] 0.37–0.71) for three-year overall survival in NSCLC and 0.37 (95% CI 0.18–0.74) in metastatic melanoma [5]. The benefit persisted in the PD-L1-negative subgroup, consistent with the prediction that vaccine-induced PD-L1 upregulation creates a therapeutic window where one did not previously exist. Meta-analytic data from earlier observational cohorts already supported the safety of mRNA vaccination in ICI-treated patients and a modestly higher rate of seroconversion than in chemotherapy-treated patients [9], making the efficacy signal a plausible biological extension.

Isolated clinical observations preceding the cohort analyses point in the same direction while removing checkpoint blockade as a confounder. Regression of metastatic salivary gland myoepithelial carcinoma followed mRNA-1273 vaccination in a patient who experienced grade 3 systemic reactogenicity, with imaging documenting tumor shrinkage of 50%, 67%, and 73% at three, six, and nine months after the second dose, and with post-vaccination biopsies showing dense infiltration by CD4+ and CD8+ T cells, natural killer cells, B cells, and dendritic cells that was absent from pre-vaccination samples [44]. Regression of multiple cutaneous melanoma metastases was likewise reported after the second BNT162b2 dose in a heavily pretreated patient, with further reduction after the third dose [45]. Neither patient was receiving an immune checkpoint inhibitor at the time of the response, so these observations isolate the vaccine-attributable component of the proposed mechanism, although the inferential weight carried by two case reports is correspondingly limited.

The Grippin observations [5] have a mechanistic precedent. Systemically administered “onion-like” multi-lamellar RNA-lipid particle aggregates (RNA-LPAs) activated RIG-I in stromal cells, producing a cytokine and chemokine cascade sufficient to reject established murine tumors [17]; a single RNA-LPA infusion also reprogrammed the tumor microenvironment within days in client-owned canines with terminal gliomas, and a first-in-human glioblastoma trial demonstrated rapid cytokine release, pseudoprogression, and glioma-specific T cell responses [17]. This work supplied the mechanistic vocabulary later applied to a clinically licensed, off-the-shelf mRNA-LNP product [5], and showed that the antigen specificity of the vaccine cargo can be uncoupled from that of the tumor when innate sensors and cDC1-mediated cross-priming are engaged in the appropriate temporal and anatomic context [5]. An orthogonal route has emerged from intratumoral administration of BNT162b2 in hosts with pre-existing anti-spike immunity, where the cargo behaves as a redirected target rather than an antigen-agnostic adjuvant [11]: intratumoral injection tagged tumor cells with mRNA-encoded spike protein and mobilized memory CD8+ T cells against the spike-displaying tumor, with partial lysis driving antigen spreading toward endogenous tumor antigens. An independent preclinical study in immunocompetent C57BL/6 mice bearing established B16F10 melanoma reported that a single intratumoral injection of the licensed COVID-19 mRNA vaccine, in the absence of pre-existing anti-spike immunity, suppressed tumor growth and prolonged survival, with subsequent doses producing additional benefit and combination with anti-PD-1 or anti-PD-L1 antibody extending both effects [46]; the mechanistic footprint included a substantial increase in intratumoral CD8+ T cell density [46]. The intratumoral and systemic routes operate through non-overlapping primary mechanisms—pre-existing pathogen-specific memory in the first, antigen-agnostic innate sensing in the second—while converging on tumor-specific T cell expansion under concurrent ICI.

The repositioning hypothesis must also be situated against personalized mRNA cancer vaccines, which provide the natural comparator class. Autogene cevumeran in resected pancreatic ductal adenocarcinoma primed polyfunctional T cell responses in approximately half of treated patients [47], with three-year follow-up confirming long-lived CD8+ T cell expansion correlating with delayed recurrence [48]. In resected melanoma, KEYNOTE-942 combined mRNA-4157 with pembrolizumab and reported an HR of 0.561 (95% CI 0.309–1.017) for recurrence-free survival, meeting the prespecified one-sided significance threshold of 0.10 [49], with a separate first-in-human study of the same platform documenting expansion of neoantigen-specific T cell clones [50]. A second-generation multi-adjuvant personal neoantigen vaccine produced potent T cell immunity in melanoma patients without dose-limiting toxicity [51], and adapted formulations directed at lung cancer neoantigens are entering clinical development to enhance ICI activity [52]. A recent review has framed personalized mRNA vaccines as the closest mechanistic analogs to the repurposing concept [10].

If a vaccine designed against SARS-CoV-2 sensitizes tumors to ICI through innate sensing rather than shared antigen specificity, the active pharmacologic principle is the LNP carrier and the modified mRNA cargo acting on innate sensors [3], not the encoded spike protein. Confirmed prospectively, this would reposition licensed COVID-19 mRNA vaccines as a class of widely deployed, low-cost antigen-agnostic immune modulators [10] with reach beyond infectious disease prophylaxis.

### 3.2. Bidirectional Dialogue Between Immune Checkpoint Inhibitors and mRNA Vaccination

The interaction between ICIs and COVID-19 mRNA vaccines runs in both directions, and the second has been established with mechanistic clarity. Patients with active malignancy, particularly those receiving cytotoxic chemotherapy, show reduced humoral and cellular responses to COVID-19 vaccination [9], a phenomenon often labeled vaccine bluntness that reflects systemic immunosuppression imposed by the tumor and by certain anti-cancer therapies [19]. In preclinical tumor-bearing models, the antibody response to a COVID-19 vaccine was severely attenuated compared with tumor-free animals, and concomitant anti-PD-1 or anti-PD-L1 antibody restored both antibody titers and cellular responses to the level observed in healthy hosts [53]. The restoration was traced to recovery of follicular helper T cell (Tfh) frequency and germinal center activity, both required for durable humoral immunity [54], and, importantly, occurred independently of the anti-tumor efficacy of the ICI, indicating that vaccine immunogenicity is improved through a pathway not contingent on tumor regression. Earlier consensus reviews of vaccination during checkpoint inhibitor therapy had already proposed this modulation, although direct experimental validation in the COVID-19 context arrived only with these preclinical models [55].

Work on TDLNs has sharpened the cellular geography of this interaction. TDLNs are enriched for tumor-specific PD-1+ T cells in close apposition with PD-L1+ conventional dendritic cells [15]; TDLN-targeted PD-L1 blockade seeded the tumor site with progenitor-exhausted T cells and improved control, whereas tumor-restricted blockade was less effective. In patients with non-metastatic melanoma, the density of PD-1/PD-L1 interactions in the TDLN, not in the primary tumor, predicted early distant recurrence, repositioning the lymph node as the principal anatomic site at which ICI acts. Because Tfh maturation and germinal center reactions occur in the same compartment, this provides the anatomic substrate for the observation that ICI rescues vaccine immunogenicity in tumor-bearing hosts. Tumor-infiltrating lymphocyte populations themselves carry biomarkers of responsiveness to combined immunomodulation, with stem-like progenitor subsets proposed as predictors of durable benefit from adoptive cellular therapy, although the evidence remains inconsistent [56].

**Figure 2:**
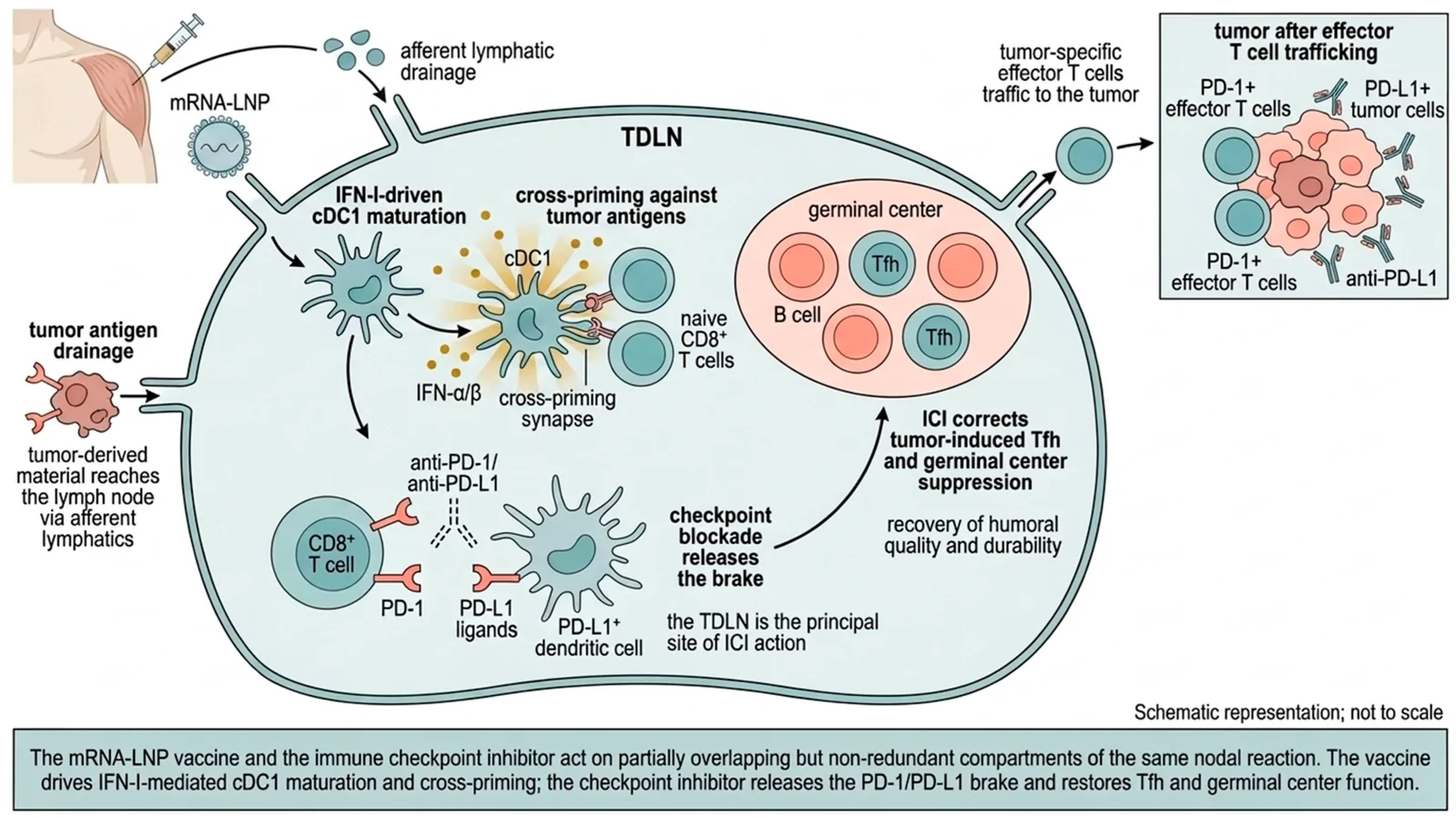
**Anatomic and cellular convergence of COVID-19 mRNA vaccine and immune checkpoint inhibitor in the tumor-draining lymph node.** Anatomic substrate within which licensed COVID-19 mRNA lipid nanoparticle (LNP) vaccines and immune checkpoint inhibitor (ICI) therapy converge in patients with malignancy. After intramuscular administration, the mRNA-LNP traffics to the tumor-draining lymph node (TDLN), where it activates the dendritic cell compartment through type I interferon (IFN-I) induction, drives cross-priming of CD8+ T cells against tumor-associated antigens, and upregulates programmed cell death ligand 1 (PD-L1) on antigen-presenting cells. The TDLN, rather than the tumor microenvironment itself, is enriched for tumor-specific programmed cell death protein 1 (PD-1)-positive T cells in close apposition with PD-L1-positive conventional dendritic cells, which establishes the lymph node as the principal anatomic site of ICI therapeutic action. Concurrent ICI administration relieves checkpoint-mediated inhibition of newly primed T cells and corrects the tumor-induced suppression of follicular helper T cell maturation and germinal center activity, enhancing the quality and durability of the vaccine-induced response. The two interventions therefore act on partially overlapping but non-redundant compartments of the same nodal reaction. Abbreviations: cDC, conventional dendritic cell; ICI, immune checkpoint inhibitor; IFN-I, type I interferon; LNP, lipid nanoparticle; mRNA, messenger RNA; PD-1, programmed cell death protein 1; PD-L1, programmed cell death ligand 1; TDLN, tumor-draining lymph node; Tfh, follicular helper T cell. *Image generated with FigureLabs.ai Scientific Illustration Software (web-based application; accessed on August 2026).*

A unified picture emerges when preclinical and clinical evidence are read together against this anatomic framework (Figure 2). The COVID-19 mRNA vaccine activates the dendritic cell compartment in the TDLN through IFN-I, cross-primes T cells against tumor-associated antigens, and upregulates PD-L1 on antigen-presenting cells [5]; ICI administered in a defined temporal window relieves checkpoint-mediated inhibition of these newly primed T cells and corrects the tumor-induced suppression of Tfh and germinal center activity [53]. The two interventions act on partially overlapping but non-redundant compartments of the same nodal reaction, consistent with the observation that their combination outperforms either alone in cold tumors where baseline T cell priming is rate-limiting. The proposal does not claim that a COVID-19 mRNA vaccine functions as a personalized tumor vaccine, because the encoded spike protein is not a tumor antigen and no clonal expansion against tumor-derived epitopes can be attributed to its direct recognition [23]; rather, the LNP and modified mRNA components act as a non-specific adjuvant whose innate effects propagate through the TDLN and, in the presence of pre-existing tumor antigens and concurrent ICI, support the priming and expansion of tumor-specific T cells.

### 3.3. Counterevidence, Methodological Discordance, and the Limits of Retrospective Inference

The discussed clinical signal is not uncontested. A retrospective cohort of 394 patients with lung cancer treated with PD-1 or PD-L1 inhibitors reported reduced immune-related arthritis and myocarditis in vaccinated patients, thyroid, dermatologic, cardiovascular, and pulmonary events unchanged, and no significant association with progression-free survival (PFS) in univariate or multivariate analyses [57]. Only 22 of the 394 patients received an mRNA vaccine, however, with the remainder receiving inactivated products (BBIBP-CorV, CoronaVac) or protein subunit formulations, so the study addresses inactivated-vaccine safety in the ICI setting rather than the mRNA-specific hypothesis. The same platform-heterogeneity caution applies to the supportive meta-analysis of vaccine response in ICI-treated patients, which also pooled products across platforms [9]. A separate Turkish cohort of 138 patients on second-line nivolumab for metastatic NSCLC found no efficacy difference between virion-based (Sinovac) and mRNA-based vaccine recipients in objective response rate, clinical benefit rate, or PFS, with grade 1–2 immune-related adverse events more frequent among Sinovac recipients [58]; the absence of an unvaccinated comparator limits inferential reach, but platform effects appear modest relative to the between-cohort heterogeneity that dominates the retrospective literature.

Three methodological features distinguish the Luo et al. [57] cohort from the supportive analyses and may contribute to divergent conclusions. Endpoints differ, with the supportive analyses evaluating median and three-year overall survival [5] and the negative report focusing on PFS [57], which is mechanistically closer to direct antitumor cytotoxicity and less sensitive to the broader immune reprogramming predicted by the antigen-agnostic model. Timing differs, with the supportive signal concentrated in patients vaccinated within 100 days of ICI initiation and the negative cohort applying no comparable temporal window. Scope differs, with the supportive cohorts spanning multiple solid tumor types and the negative cohort restricted to lung cancer; earlier single-institution data in melanoma diagnosed during the pandemic reported COVID-19 severity and mortality by variant period without an association with melanoma stage or treatment, which constrains the extent to which pandemic-era disease trajectories can explain the vaccine signal [59]. If the magnitude of benefit varies by tumor type, restricting the analysis to a single malignancy may have attenuated the observed association.

Two prospective studies have refined the picture. After two doses of BNT162b2 or ChAdOx1-S, NSCLC patients on ICI showed lower and more variable serum IgG titers against the spike receptor-binding domain and lower interferon-γ T cell responses than controls, and a third dose of BNT162b2 raised both to the level of controls with humoral and cellular readouts correlating significantly [20]; the relevant immunization is therefore the boosted regimen, and analyses restricted to two-dose recipients underestimate the immunological footprint of vaccination in this population. The phase IV CoVigi trial extended this across 204 patients with solid cancer and 73 controls: among SARS-CoV-2-naïve patients, 65% developed anti-spike antibodies after the first dose, 92% after the second, and 100% by six months, with chemotherapy the only pharmacologic class that impaired both humoral and cellular responses [60]. CoVigi therefore repositions chemotherapy, not ICI, as the dominant pharmacologic determinant of vaccine bluntness.

The pre-COVID literature on vaccination in ICI-treated patients had focused on seasonal influenza vaccines, which were shown to be safe and to elicit protective titers without amplifying immune-related adverse events [61]. Vaccinated patients with lung cancer were likewise not at increased risk of severe vaccine-related events relative to the general population [62]. These observations supply the baseline safety precedent against which the COVID-19 vaccination experience in this setting can be calibrated.

The observational base underlying the vaccine-ICI hypothesis has since expanded beyond the original MD Anderson analysis. Five additional retrospective cohorts, spanning cancer centers and national registries in Israel, France, Bulgaria, Turkey, and the multinational TriNetX Research Global Network, have examined the association between peri-ICI COVID-19 mRNA vaccination and clinical outcomes (Table 1). Their designs, populations, endpoints, and effect estimates are reported in full in Table 1 and are not restated here. Two features of the set are relevant to the interpretation that follows. The cohorts differ substantially in endpoint definition, ranging from overall survival in the French and Israeli analyses [39,43] to long-term mortality after COVID-19 hospitalization in the Bulgarian registry [42] and progression-free survival on subsequent therapy in the Turkish cohort [41], so the reported estimates are not directly poolable. The largest of them applied target trial emulation to 155,300 ICI-treated patients and reported a 14% reduction in all-cause mortality and a 57% reduction in intensive care unit admission after propensity score matching [40]. Taken together, the six retrospective analyses converge on a directional survival advantage in the vaccinated stratum across geographically and methodologically heterogeneous populations, with reported hazard reductions ranging from approximately 14% in the target trial emulation [40] to 49% in the original MD Anderson NSCLC cohort [5].

A methodologically rigorous re-analysis has however challenged the observational signal at its foundation. Jee and colleagues studied 8368 patients treated with ICI at Memorial Sloan Kettering (MSK) Cancer Center between 2017 and 2022 and identified three findings that materially attenuate the causal interpretation of the vaccine-ICI association [65]. Applying the original Grippin framework [5] reproduced the survival association, but stratification by calendar year confined the effect to patients starting ICI in 2021, coincident with peak pandemic-era masking and quarantining, with the signal dissipating in 2022; a parallel re-analysis of the MD Anderson NSCLC data yielded hazard ratios approaching 1.0 for patients treated in 2022. Extension to non-ICI antineoplastic agents (5-fluorouracil, osimertinib) reproduced the same temporal pattern, indicating that the peri-vaccination survival benefit was not specific to ICI. Landmarking at 100 days after ICI initiation and restricting to March 2021 onward, when vaccination had been broadly available, abolished the survival difference, whereas the standard analytic approach retained a significant benefit. The convergence of temporal restriction, absence of ICI specificity, and disappearance under landmark analysis is difficult to reconcile with a mechanistically driven priming effect and is consistent with selection bias favoring vaccination in patients with more favorable prognoses during the early pandemic.

Two considerations qualify but do not overturn the MSK critique. The multi-country replications comprised calendar years and health-system contexts distinct from those in which early-pandemic selection bias would have been strongest; the Chen target trial emulation in particular included patients treated through December 2025 and detected a stronger mortality benefit in the 2023–2025 stratum (HR 0.83) than in 2021–2022 (HR 1.02), a pattern inconsistent with a purely early-pandemic selection effect [40]. The biological plausibility of the antigen-agnostic model does not rest on the retrospective association alone, because IFN-I-driven dendritic cell activation, CD8+ T cell cross-priming and PD-L1 upregulation have been documented both in murine tumor models and in healthy human volunteers receiving licensed COVID-19 vaccines [5]. The Jee re-analysis [65] established that the magnitude of the association in the original MD Anderson cohort likely overstates the true causal effect; it did not establish that the effect is null, and the residual uncertainty maps directly onto the case for prospective evaluation.

Complementary safety data available at the time of those analyses indicated that mRNA vaccination in ICI-treated patients did not amplify immune-related adverse events and that thromboembolic risk after a third dose was not increased in aggregate, with a natural killer cell response pattern acting as an independent stratifier rather than as a contraindication [9,63]. The immunometabolic state of the host nonetheless shapes both efficacy and rare adverse events.

A coherent interpretation emerges from these findings. Almost all efficacy studies to date are retrospective, and heterogeneity in vaccine platform, endpoint, timing window, dose number and tumor type accounts for much of the observed variability. The single prospective study, the Vax-On-Third analysis of 226 patients with metastatic NSCLC on first-line pembrolizumab or cemiplimab, detected no overall improvement in progression-free or overall survival with periodic mRNA boosters after propensity-score matching, with a positive signal restricted to the PD-L1 ≥50% single-agent immunotherapy subgroup (HR 0.59, 95% CI 0.36–0.96, *p* = 0.034) [64]; the direction is opposite to the Grippin observation of benefit in PD-L1-negative disease, and this discordance indicates that the retrospective literature has not resolved which subphenotype is most sensitive to mRNA-driven priming. The magnitude of the earliest hazard reductions approaches that attributable to checkpoint inhibitors themselves, difficult to reconcile with a transient adjuvant effect and raising the prior probability that immortal-time and healthy-vaccinee effects contribute materially to the association. Taken with the Jee re-analysis [65], the more conservative estimates from the multi-country replications [39,40,41,42,43] are consistent with a true effect of moderate magnitude superimposed on a larger apparent effect driven by residual confounding. The biological signal remains plausible, being supported by IFN-I, dendritic cell activation and PD-L1 upregulation in cold tumors; it appears fragile, being detectable only within a defined temporal window relative to ICI initiation and when the endpoint reflects durable immune control. Whether it withstands prospective evaluation remains the key question.

### 3.4. Candidate Clinical Contexts for Prospective Evaluation

If the antigen-agnostic model is correct, the populations most likely to benefit are those in which ICI monotherapy underperforms because a cold tumor microenvironment, with low T cell infiltration, low tumor mutational burden and low PD-L1 expression, is rate-limiting [38]. Survival gains reported within the PD-L1-negative subgroup in retrospective cohorts [5] provide a starting point for candidate selection. The systemic intramuscular route is the most directly translatable option, whereas intratumoral delivery in patients with accessible disease constitutes a complementary configuration warranting dedicated evaluation [11].

The strongest candidate is PD-L1-negative NSCLC. The population is large, ICI plus chemotherapy is the standard of care on the basis of randomized evidence [33], and a substantial fraction of patients still fail to respond. Neoadjuvant and perioperative pembrolizumab-based regimens have yielded event-free survival gains in resectable NSCLC [66], and long-term follow-up of neoadjuvant nivolumab plus chemotherapy has now demonstrated sustained overall survival benefit [67]. The retrospective signal detected across PD-L1 strata in lung cancer [5], with the mechanistic prediction that vaccine-induced PD-L1 upregulation creates a therapeutic window precisely where baseline PD-L1 is lowest, makes this subgroup the most informative test bed. Cost-effectiveness analyses of second-line nivolumab in NSCLC have reported incremental cost-effectiveness ratios that strain payer thresholds in the health systems evaluated [68], with comparable findings for first-line pembrolizumab and dostarlimab [69]; a repurposed adjuvant costing orders of magnitude less than ICI, requiring no new infrastructure, would substantially improve the cost-effectiveness profile of any ICI-based regimen in which it produced incremental survival.

Pancreatic ductal adenocarcinoma represents a second candidate. ICI monotherapy has consistently failed in unselected pancreatic adenocarcinoma populations; the cold immunogenic profile, the abundant desmoplastic stroma, and the failure of dual checkpoint blockade to reach its efficacy threshold in a randomized phase 2 trial [70] make pancreatic cancer a demanding but mechanistically interpretable setting [71]. A positive signal in this disease would be highly informative; a negative signal would still be interpretable, because the cold phenotype is so consistent that population heterogeneity would not confound the result. Personalized mRNA neoantigen vaccines have already documented immunogenicity in this setting [47], supplying both a mechanistic comparator and a logistically distinct strategy to which the repurposing approach must be measured.

Two additional candidates deserve consideration. Microsatellite-stable colorectal cancer accounts for most metastatic colorectal cancer and is unresponsive to ICI monotherapy, in contrast to the small microsatellite-instability-high subgroup; the population is sufficiently large for adequately powered trials and the cold phenotype is well characterized. Pleural mesothelioma is a smaller but accessible population in which ICI combinations have been approved, with chronic inflammation, fibrotic stroma and limited T cell infiltration all expected to benefit from a systemic IFN-I-driven priming signal. A review of next-generation mRNA vaccine platforms has highlighted these settings as priority indications for combined immunomodulatory development [72], and a recent viewpoint has framed the integration of therapeutic vaccines with immune checkpoint blockade as a priority direction [73]. Selected sarcomas and high-grade glioma may also warrant exploration in light of the canine and first-in-human glioblastoma data, although heterogeneity within each category will demand careful stratification.

Among these oncologic settings, PD-L1-negative NSCLC is carried forward as the priority oncologic context in the five-setting prospective framework proposed here, with pancreatic, microsatellite-stable colorectal and mesothelial disease retained as secondary candidates for subsequent evaluation. A practical consideration distinguishes the repurposing question from conventional combination drug development: the vaccine is already approved, produced at scale, distributed worldwide, and supported by a population-level safety database that no investigational adjuvant could match, at a cost per dose that is low relative to ICI and with no new infrastructure. Operational readiness for a definitive prospective trial is therefore favorable. The remaining challenges are scientific—the optimal temporal window relative to ICI initiation, the choice between primary series and boosted regimens informed by recent prospective immunogenicity data [20], efficacy endpoints sensitive to delayed immune-mediated benefit, and the management of competing inflammatory and thromboembolic risks.

## 4. Beyond Oncology

If the LNP carrier and modified mRNA cargo act as antigen-agnostic immunomodulators [5], the repurposing case extends to any clinical context where coordinated innate activation, IFN-I signaling or relief of immune exhaustion has therapeutic value. Beyond oncology, four further entities meet two stringent criteria simultaneously, a mechanistically validated biological rationale and an established pharmacologic precedent: perioperative immune dysfunction in major cardiac surgery, chronic hepatitis B virus (HBV) infection with unmet need for functional cure, human immunodeficiency virus (HIV) reservoir persistence and T cell exhaustion under suppressive antiretroviral therapy, and sepsis-induced immunoparalysis. Other domains were excluded because the rationale is weaker, the unmet need has been met by alternative therapeutics, or the proposed mechanism is incompatible with the immunometabolic state of the target population.

### 4.1. Perioperative Immune Dysfunction in Major Cardiac Surgery

Major cardiac surgery induces a stereotyped transient immunosuppression that predisposes to post-operative sepsis, acute kidney injury (AKI), ventilator-associated pneumonia (VAP), and prolonged intensive care unit stay [74]. The biological substrate involves reduced monocyte responsiveness, attenuated antigen presentation, and an inflammatory cytokine pattern that compromises both pathogen clearance and tissue repair. Strategies intended to restore immune competence in this setting have been reviewed, and none of the approaches evaluated to date has established a pharmacologic means of priming innate immunity before surgery [74].

The conceptual precedent comes from trained immunity research with Bacillus Calmette-Guérin (BCG), the only routinely administered vaccine for which non-specific protective effects against unrelated pathogens have been established in randomized trials [75]. Trained immunity reflects durable functional reprogramming of monocytes, natural killer cells and hematopoietic stem and progenitor cells, mediated by histone modifications at promoters of inflammatory genes and by metabolic rewiring toward aerobic glycolysis [76,77], and has been formalized as a consensus immunological category distinct from tolerance, priming and conventional differentiation [78] and positioned as a tractable lever in immunopathology and transplantation [79]. Pre-clinical work on the BCG-induced phenotype detailed the role of myeloid progenitors and epigenetic memory in defining the duration and quality of innate protection [77]. The randomized BRACE trial in health care workers showed that BCG vaccination did not significantly reduce symptomatic COVID-19, but validated the operational feasibility of randomized trained-immunity evaluation [80]. The precedent establishes that a vaccine designed for one pathogen can produce non-specific protection through trained immunity, independent of the encoded antigen; safety reviews of BCG revaccination further support the feasibility of repeated trained-immunity-inducing exposures in adult populations [81].

mRNA-LNP vaccines induce a signature compatible with the trained-immunity phenotype in human peripheral blood, although durable epigenetic reprogramming of hematopoietic stem and progenitor cells of the kind described for BCG has not been formally demonstrated in humans. Systems vaccinology of BNT162b2 recipients identified expansion of CD14+CD16+ inflammatory monocytes with interferon-response transcription factor signatures after the second dose [4], features associated with the BCG-induced trained-immunity phenotype and consistent with partial convergence across antigenically unrelated vaccine platforms [82]. Pre-operative administration would therefore generate a window of innate immune competence at the time when surgical immunosuppression is most pronounced [83], potentially mitigating post-operative infection, AKI and prolonged ventilator dependence.

A pragmatic trial can specify pre-operative administration of a standard mRNA-LNP vaccine dose seven to fourteen days before elective coronary artery bypass grafting or valve surgery, with co-primary endpoints of post-operative sepsis (Sepsis-3) and stage ≥ 2 AKI within 30 days, and secondary endpoints including VAP incidence, intensive care unit length of stay, 30-day mortality and inflammatory biomarker trajectories. The intervention is logistically tractable, the safety profile is established [1,9], and the patient population is large and well-defined within existing cardiac surgical programs. Co-administration with seasonal influenza vaccines preserves the immunogenicity of both products at the cost of a modest increase in reactogenicity, providing a regulatory precedent for combining immunomodulatory vaccines around the surgical event [84]. Alternative approaches to perioperative immunomodulation are limited and dominated by negative or inconclusive trials of immunonutrition and selective beta-blockade, leaving the trained-immunity mRNA-LNP approach as a mechanistically distinct candidate warranting direct prospective evaluation.

### 4.2. Hepatitis B Chronic Infection and Functional Cure

Chronic HBV infection persists in more than 250 million individuals worldwide despite the availability of safe nucleos(t)ide analog suppressive therapy, with functional cure defined by sustained loss of hepatitis B surface antigen remaining elusive in the vast majority of treated patients. The therapeutic gap between viral suppression and functional cure has motivated systematic exploration of host-directed antiviral strategies, with particular focus on innate immune activation through TLR7 and TLR8 signaling [85].

The rationale for TLR7/8 engagement in chronic HBV builds on the antiviral activity of pegylated interferon-alpha, the only standard-of-care agent producing sustained off-treatment responses in a minority of patients, through IFN-α-mediated suppression of viral transcription, natural killer cell activation and reinvigoration of exhausted hepatitis-specific CD8+ T cells in the liver [85,86]. Direct TLR7 agonists are in active clinical development as orally bioavailable alternatives: the first-in-human phase 1 trial of JNJ-64794964 showed dose-dependent induction of interferon-stimulated gene expression, IFN-α release, CXCL10 elevation and natural killer and B cell activation in healthy volunteers [85], and the hepatotropic TLR7 agonist SA-5 reinvigorated exhausted CD8+ T cells and recruited macrophages with concomitant viral suppression in a chronic HBV mouse model [86]. This pipeline validates the host-directed approach and defines the immunopharmacologic profile sought in adjunctive intervention.

The repurposing case for COVID-19 mRNA vaccines in chronic HBV rests on partial convergence of these mechanisms. mRNA-LNP vaccines engage TLR7 and TLR8 through the single-stranded RNA cargo, although nucleoside modification with N1-methylpseudouridine attenuates this signaling substantially [2,18]; the residual sensor activity nonetheless generates a systemic IFN-I response [5] together with activation of natural killer cells and innate compartments at scale [3]. The pharmacologic signature therefore overlaps partially with the profile sought by purpose-designed TLR7 agonists, with the caveat that the dose-titratable, sustained TLR7 activation of an oral agonist cannot be replicated by a fixed intramuscular dose; the platform offers instead the advantage of prior approval, industrial-scale manufacturing and extensive safety data [1]. A pragmatic trial would evaluate adjunctive mRNA-LNP vaccination in patients on stable nucleos(t)ide analog therapy with persistent hepatitis B surface antigen positivity, with primary endpoint of hepatitis B surface antigen loss at 96 weeks and a key secondary endpoint of quantitative decline of one log10 or greater, calibrating dose, schedule and baseline-titer stratification against the TLR7 agonist development program.

Clinical observations in chronic HBV provide preliminary support that bears directly on the proposed endpoint. Rapid reduction of hepatitis B surface antigen was documented after COVID-19 vaccination in a series of patients with chronic HBV infection under nucleos(t)ide analog therapy [87]. An interrupted time series analysis subsequently identified a temporal association between COVID-19 vaccination and hepatitis B surface antigen loss in a chronic hepatitis B cohort, extending the case observations to a population-level design [88]. Neither study was randomized and both remain susceptible to secular trend and ascertainment effects, although together they establish that the endpoint proposed here has been observed rather than merely postulated.

### 4.3. HIV Chronic Infection and T Cell Exhaustion Under Suppressive Therapy

Chronic HIV infection under suppressive antiretroviral therapy (ART) is characterized by persistent immune dysregulation, with residual IFN-I signaling, microbial translocation and a T cell exhaustion phenotype resembling that of tumor-bearing hosts [89]. The HIV reservoir, largely composed of resting memory CD4+ T cells expressing PD-1 and cytotoxic T lymphocyte-associated protein 4 (CTLA-4), persists despite suppressive therapy and re-establishes viremia upon interruption [90]. The convergence of these features with the immunology of cancer has motivated pilot studies of immune checkpoint blockade in people living with HIV, with attention to latency reversal and reservoir contraction; contemporary reviews frame the persistent reservoir as the central obstacle to therapeutic eradication and identify combinatorial immunomodulation as the most plausible path toward functional cure [91].

The AIDS Malignancy Consortium 095 study evaluated nivolumab with or without ipilimumab in 40 antiretroviral-suppressed people living with HIV (PWH) and concurrent advanced malignancies [90]. Dual PD-1 and CTLA-4 blockade produced a 1.44-fold increase in cell-associated unspliced HIV RNA after the first dose, with viral outgrowth assay decreases of 97% and 64% in the two participants with sequential samples, consistent with latency reversal and partial reservoir contraction. In simian immunodeficiency virus-infected rhesus macaques on suppressive therapy, dual blockade induced pronounced viral reactivation and decreased intact viral DNA in lymph node B cell follicles, although reservoir contraction remained insufficient to alter post-treatment viral control [92]. Other reservoir-targeting strategies under development include CRISPR-Cas approaches to excise proviral DNA [93] and targeted activators of cell kill, which selectively eliminate HIV-1 expressing cells by accelerating Gag-Pol dimerization and triggering premature activation of the viral protease [94]; each depends on first reactivating the latent reservoir, a step at which mRNA-LNP innate activation may contribute.

Direct evidence that mRNA-LNP vaccination engages the latent reservoir has since been reported. SARS-CoV-2 mRNA vaccination exposed latent HIV to Nef-specific CD8+ T cells in antiretroviral-suppressed participants, demonstrating that the innate activation delivered by the platform is sufficient to render reservoir-harboring cells visible to pre-existing HIV-specific effectors [95]. This observation supplies the mechanistic step that the repurposing case in this setting would otherwise have to assume.

mRNA-LNP vaccination preserves humoral and cellular responses in people living with HIV with adequate CD4 counts [96], establishing the safety baseline for adjunctive use. The repurposing case rests on three propositions: the vaccine-induced IFN-I cascade may partially reverse residual immune exhaustion through the mechanisms operative in the oncologic setting [5]; TLR7/8 stimulation may amplify host antiviral programs in the residual HIV reservoir [95]; and mRNA-LNP innate reprogramming may potentiate the modest latency reversal observed with immune checkpoint blockade alone. A pragmatic trial would evaluate mRNA-LNP vaccination, with or without concurrent low-dose anti-PD-1, in antiretroviral-suppressed adults with stable viral suppression and adequate CD4 reconstitution, with endpoints of cell-associated unspliced HIV RNA, intact proviral DNA and viremia rebound timing under analytical treatment interruption. Safety considerations specific to immune checkpoint blockade in people living with HIV require dose stratification and careful monitoring of immune-related adverse events [97].

### 4.4. Sepsis-Induced Immunoparalysis

Late-phase sepsis is characterized by a sustained immunosuppressive state that persists after the acute inflammatory phase has resolved, with attenuated monocyte HLA-DR expression, lymphopenia, exhausted T cell phenotypes, and impaired pathogen clearance that together drive secondary infections and mortality [98]. The condition has no approved pharmacologic therapy, and the failure of broad anti-inflammatory strategies across two decades of trials reflects the recognition that the late phase requires immune restoration rather than further suppression.

The pharmacologic precedent for the immune-restoration strategy comes from the PROVIDE randomized trial, which used biomarker-guided personalized immunotherapy in patients with sepsis-induced immunoparalysis. Patients identified by ferritin elevation (macrophage activation syndrome phenotype) received anakinra, while those identified by reduced monocyte HLA-DR expression (immunoparalysis phenotype) received recombinant interferon-γ; clinical improvement at day 7 was observed in the anakinra-treated macrophage activation syndrome stratum, whereas only two patients met immunoparalysis criteria and the trial did not meet its primary endpoint [99]. The trial established that biomarker stratification can identify immunometabolic phenotypes amenable to targeted immunomodulation in sepsis, defining the operational template against which any new candidate must be measured.

The mechanistic case for mRNA-LNP vaccines in sepsis-induced immunoparalysis rests on partial alignment between the vaccine-induced IFN-I signature and the host signaling deficit observed in the immunoparalysis phenotype. Systems vaccinology data have documented a marked IFN-response transcriptional signature after the second BNT162b2 dose, with expansion of monocyte populations expressing interferon-response transcription factors [4]. The recombinant IFN-γ used in PROVIDE engages the type II interferon receptor and drives monocyte HLA-DR restoration more directly than type I interferons do, so an mRNA-LNP intervention should be regarded as a mechanistic complement rather than an equivalent pharmacologic replacement. A further consideration is that type I interferon exerts context-dependent and potentially deleterious effects in sepsis, having been implicated experimentally in lymphocyte apoptosis and in the monocyte dysfunction that characterizes immunoparalysis; whether a vaccine-induced type I interferon pulse would restore or further impair innate competence in late-phase sepsis is therefore genuinely uncertain, and this uncertainty, not logistical simplicity, should govern whether the setting is prioritized for early testing. Prospective evaluation should include HLA-DR restoration as an early pharmacodynamic marker before mortality endpoints are relied upon. A pragmatic trial would enroll patients with persistent immunoparalysis at 72 h after sepsis onset, identified by monocyte HLA-DR below the established threshold, and randomize to a single dose of mRNA-LNP or standard care, with co-primary endpoints of monocyte HLA-DR restoration at 96 h and 28-day mortality, and secondary endpoints of secondary infection incidence, ventilator-free days and inflammatory biomarker trajectories. The intervention is logistically simple, can be delivered at the bedside, and benefits from the same population-level safety database as the elective surgical setting [1].

## 5. Safety in the Proposed Repurposing Settings

### 5.1. Combination with Immune Checkpoint Inhibitors in Oncology

In ICI-treated patients, mRNA-LNP vaccination has been evaluated in 8331 individuals across 27 studies, of whom 4724 received ICI [9]. irAE incidence was 21.96% before vaccination and 14.88% afterward, with no signal of vaccine-induced amplification of immune toxicity; seroconversion in ICI-treated patients was marginally higher than in chemotherapy-treated patients (RR 1.05, 95% CI 1.01–1.10, *p* = 0.02) and did not differ significantly from healthy controls. The phase IV CoVigi trial extended these observations to 204 patients with solid cancer, with chemotherapy the principal pharmacologic suppressor of vaccine response and ICI, monoclonal antibody, tyrosine kinase inhibitor and curative radiotherapy preserving T cell responsiveness [60].

The Vax-On-Third-Profile study of 429 patients with cancer found that no treatment category remained an independent predictor of venous thromboembolism after a third BNT162b2 dose in multivariate analysis, with a distinctive natural killer cell response pattern independently associated with thromboembolic events [63]. A retrospective cohort of 394 patients on PD-1 or PD-L1 inhibitors reported reduced immune-related arthritis and myocarditis in vaccinated patients, without amplification of other irAE categories [57]. The existing irAE monitoring framework captures these signals without protocol modification [9].

The target trial emulation of 7218 propensity-matched ICI-treated patients from the TriNetX Research Global Network refined the safety profile with quantitative precision [40]. The composite incidence of any-grade irAE was 22% higher in the vaccinated stratum at 36 months (HR 1.22, 95% CI 1.17–1.28), with an 11% increase in severe irAE (HR 1.11, 95% CI 1.04–1.18); organ-specific analysis identified ocular manifestations as the principal driver (HR 1.48, 95% CI 1.17–1.87), the only organ system to retain statistical significance after Benjamini–Hochberg correction, persisting after exclusion of events within 90 days of ICI initiation. In the same cohort, all-cause mortality was 14% lower (HR 0.86, 95% CI 0.80–0.93) and intensive care unit admission 57% lower (HR 0.43, 95% CI 0.25–0.73), indicating that the incremental irAE burden did not translate into worse net outcomes. Ophthalmological monitoring is therefore the surveillance addition most likely to have clinical impact.

### 5.2. Perioperative Cardiac Surgery Setting

Post-vaccination myocarditis is the most consistently replicated mRNA-LNP safety signal, with absolute incidence of approximately 5–11 excess cases per 100,000 second doses concentrated in males aged 16–24 years [100]. Risk after sequential doses has been stratified by age and sex and differs by product, since BNT162b2 incidence rate ratios did not decline at the booster dose whereas the mRNA-1273 signal attenuated [101,102]. The population undergoing elective coronary artery bypass grafting or valve surgery lies outside the affected demographic, since the excess is restricted to individuals below 40 years whereas the median age at cardiac surgery exceeds 60 years [102]; myocarditis after SARS-CoV-2 infection in unvaccinated young adults exceeds the post-vaccine risk with relative risks between 1.8 and 5.6 [103]. Large-scale evidence on cardiovascular outcomes after COVID-19 vaccination has not identified an excess of acute myocardial infarction or stroke at population level [104], whereas a Bayesian multivariate meta-analysis reported an association with coronary artery disease that awaits independent replication [105].

Cytokine elevations from vaccine-induced innate activation and from the cardiopulmonary bypass inflammatory cascade may converge on the myocardium during the early post-operative window [106]. A pre-operative interval of seven to fourteen days allows the acute innate response to resolve before surgical insult, with trained-immunity reprogramming persisting beyond, consistent with the kinetics of innate training documented in humans [76,107]. Peri-operative troponin monitoring is standard in cardiac surgical care and provides adequate surveillance for vaccine-attributable cardiac inflammation without additional protocol burden [108]. A complementary rationale for incorporating COVID-19 vaccination into cardiovascular preventive care has been articulated in cardiology reviews [109].

### 5.3. Chronic Hepatitis B Treatment Setting

Pegylated interferon-alpha therapy in chronic HBV is associated with hepatic flares reflecting immune-mediated hepatocyte killing during viral antigen clearance, a proportion of which can be clinically significant [110]. The first-in-human phase 1 trial of the oral TLR7 agonist JNJ-64794964 reported flu-like symptoms at higher exposure levels and no severe hepatic events in healthy volunteers, with TLR7-mediated IFN-α induction confined to the dose range tolerable for chronic administration [85]. Population-scale pharmacovigilance on mRNA-LNP vaccination has not identified an excess among the adverse events of special interest assessed [1], although hepatic endpoints were not among those evaluated and the question remains open in patients with chronic liver disease. Pre-specified alanine aminotransferase thresholds and monitoring intervals adapted to the kinetics of immune-mediated hepatic flares provide the operational framework for a pragmatic trial of mRNA-LNP as adjunct to nucleos(t)ide analog therapy.

### 5.4. HIV Chronic Infection and Combination with Checkpoint Blockade

Anti-PD-1 monoclonal antibody with or without anti-CTLA-4 was well tolerated in PWH on ART with concurrent advanced malignancies in the AIDS Malignancy Consortium 095 study, in which 36 participants received nivolumab and grade ≥ 3 immune-related adverse events occurred in 14% [111], with no unexpected toxicity attributed to chronic HIV infection and a 1.44-fold increase in cell-associated unspliced HIV RNA without clinical decompensation. In simian immunodeficiency virus-infected rhesus macaques on suppressive therapy, dual checkpoint blockade was well tolerated even at doses sufficient to induce viral reactivation [92]. Immunogenicity in this population is CD4-dependent, with responses in participants above 500 cells/mm^3^ comparable to HIV-negative controls [96]. Analytical treatment interruption protocols, used as endpoint for reservoir studies, carry intrinsic safety considerations independent of any vaccine intervention and require pre-specified viral load thresholds for ART resumption.

The safety profile of mRNA-LNP vaccination in this population is documented across systematic reviews and prospective cohorts. A meta-analysis of 34 studies covering 4517 PWH reported seroconversion and adverse event rates comparable to healthy controls after either dose of the primary series [112], with a second independent meta-analysis reaching concordant conclusions on immunogenicity and tolerability [113]. Booster dosing has been examined specifically, with 54 studies documenting no serious adverse events attributable to the third or subsequent dose and seroconversion rates comparable to controls, while the magnitude of the response remained dependent on CD4 count [114]. Prospective follow-up of PWH on ART receiving heterologous booster regimens recorded no severe vaccine-related adverse events irrespective of CD4 stratum, alongside increases in CD4 and CD8 T cell counts and a decline in cell-associated HIV RNA after three doses [115].

### 5.5. Sepsis-Induced Immunoparalysis Setting

Patients enrolled in immunomodulatory sepsis trials carry baseline mortality risks that dwarf the safety signal of any individual immunotherapy, and PROVIDE showed that biomarker-guided personalized immunotherapy was tolerated in a critically ill cohort without unexpected adverse events [99]. The proposed mRNA-LNP intervention is administered as a single bedside dose in patients already receiving intensive supportive care, with relevant safety considerations relating to the kinetics of inflammatory response rather than chronic exposure. Pre-specified troponin, C-reactive protein and procalcitonin monitoring adapted to the sepsis-acute care setting capture cardiovascular and inflammatory signals without additional infrastructure.

### 5.6. Population-Scale Safety Database

The safety case for any repurposing trial is anchored by the pharmacovigilance evidence accumulated since deployment. Sequential-dose surveillance has not signaled an increase in severe adverse events beyond those already characterized, and the US bivalent vaccine surveillance program documented no new safety signals beyond those characterized in the primary series [116]. COVID-19 vaccination during 2020–2024 averted an estimated 2.5 million deaths worldwide and 14.8 million life-years across all formulations, providing the denominator against which any repurposing risk must be calibrated [117]. Systematic review of incident systemic autoimmune rheumatic disease after mRNA vaccination has not indicated a consistent increase [118], and autoantibody profiling after heterologous boosting has detected no consistent pattern of post-vaccination autoimmune induction [119]; the established exceptions remain myocarditis and the platform-specific signals identified in large-scale surveillance [1].

A small set of editorial reports has raised hypothetical concerns that warrant explicit acknowledgement. Postural orthostatic tachycardia syndrome has been reported following both SARS-CoV-2 infection and vaccination, with systematic review identifying infection as the dominant antecedent and vaccination contributing a substantially smaller fraction [120]. Experimental and population data indicate that respiratory viral infection, rather than vaccination, can awaken dormant cancer cells, since influenza and SARS-CoV-2 infection drove loss of the pro-dormancy phenotype in disseminated breast carcinoma cells with confirmation in cancer survivor cohorts [121], a distinction that accompanying editorial commentary framed in terms of infection as the relevant exposure [122]. A separate report proposed an association between COVID-19 mRNA vaccination and increased incidence of Alzheimer’s disease and mild cognitive impairment in a cohort drawn from a 50% random sample of Seoul residents aged ≥65 years [123], but the finding has not been replicated in comparably powered independent cohorts, and the adverse events of special interest assessed in population-scale pharmacovigilance were acute immune-mediated neurological events rather than neurodegenerative endpoints [1].

The distinction between post-acute sequelae of COVID-19 infection and post-vaccination syndromes is central to safety interpretation, with overlapping symptomatology but biological substrates that have been proposed as distinct rather than demonstrated to be so [124]. Vaccination reduces the risk of developing long COVID after subsequent SARS-CoV-2 infection [125], and updated syntheses have quantified the infection-attributable burden against which that reduction is measured [126].

### 5.7. Autoimmune Risk Intrinsic to the Proposed Mechanism

The mechanism proposed here carries a symmetrical risk that requires explicit treatment. If robust innate activation broadens cross-presentation of locally available antigens, then self-antigens released during inflammation or tissue injury become available to the same cDC1 compartment that presents tumor-derived material. In individuals harboring pre-existing autoreactive clones, and particularly under concurrent checkpoint blockade in which peripheral tolerance is already attenuated, that broadened presentation could in principle license autoreactive T cells rather than tumor-specific ones. The pathogenesis of immune-related adverse events under ICI therapy is consistent with this concern, because it is attributed in part to the unmasking of self-reactivity that peripheral tolerance normally restrains [127].

Three considerations bound the magnitude of that risk without eliminating it. First, the innate stimulus delivered by a single intramuscular mRNA-LNP dose is transient, because the local response peaks within 24 h and resolves over the following days [3,4], whereas the experimental systems in which determinant spreading drives autoimmunity depend on sustained or repeated antigenic challenge within an inflamed target organ [27]. Second, the vaccine does not itself produce tissue injury at the sites where autoreactive priming would matter, so the release of previously sequestered self-antigen that initiates spreading in autoimmune models has no evident counterpart in this setting. Third, the empirical record accumulated across billions of doses does not display the signal that the mechanism would predict if it operated at clinically relevant scale, because systematic review of incident systemic autoimmune rheumatic disease after mRNA vaccination has not indicated a consistent increase [118], meta-analysis of ICI-treated patients has not documented amplification of immune-related adverse events after vaccination [9], and a retrospective cohort of patients receiving PD-1 or PD-L1 inhibitors reported reduced rather than increased incidence of immune-related arthritis and myocarditis [57].

The residual uncertainty is nonetheless sufficient to shape trial design rather than to be set aside. Prospective protocols should exclude patients with active autoimmune disease requiring systemic immunosuppression and those with a history of grade 3 or higher immune-related adverse events, both of which are standard exclusions in checkpoint blockade trials and were applied in the phase 1 assessment of pembrolizumab in people living with HIV [97]. Surveillance should be weighted toward the interval during which vaccine-induced IFN-I induction overlaps with checkpoint-released T cell activity, with organ-specific monitoring for thyroiditis, hepatitis, colitis, and myocarditis at the visits immediately following vaccination. Autoantibody profiling at baseline and at defined post-vaccination timepoints would convert a theoretical concern into a measurable endpoint, and pre-specifying it as a secondary safety outcome would allow the mechanism to be tested rather than assumed. Administering a single dose at a defined interval relative to ICI initiation, rather than repeated dosing during active checkpoint therapy, further limits the window during which the two stimuli coincide.

## 6. Methodological Framework for Causal Inference

Causal inference for mRNA-LNP repurposing rests on three complementary methodological frameworks, each addressing a distinct source of bias in the path from retrospective signal to confirmatory evidence.

### 6.1. Target Trial Emulation

Target trial emulation is a framework for designing observational analyses that explicitly approximate the protocol of a hypothetical randomized trial [128]. The framework specifies eligibility criteria, treatment strategies, time-zero alignment, follow-up timing, and the causal contrast intended for estimation. The explicit goal is to avoid the immortal time bias and confounding by indication that have historically distorted observational comparisons of treatment strategies in clinical epidemiology [129]. Specifying the target trial protocol before any data analysis prevents immortal time bias and other self-inflicted methodological injuries that emerge when the analytic design is constructed in parallel with the data exploration [130]. The retrospective oncologic signal that motivates the present analysis illustrates both the value and the limits of the approach. Vaccine exposure within 100 days of ICI initiation was not randomized, and the decision to vaccinate may have been influenced by performance status, expected survival, and access to care [5]. The E-value sensitivity statistic quantifies the magnitude of unmeasured confounding required to nullify the observed association, providing a transparent threshold against which residual bias can be evaluated. The discrepancy between the survival signal in supportive cohorts and the negative PFS result in lung cancer [57] is consistent with the residual confounding that the framework is designed to surface and quantify.

### 6.2. Mendelian Randomization

Mendelian randomization uses germline genetic variants as instrumental variables for exposure, exploiting their random assortment at meiosis to address confounding from environmental and lifestyle factors [131]. The method rests on three assumptions, namely instrument relevance, instrument independence, and the exclusion restriction. Curated platforms with broad coverage of genome-wide association results and standardized two-sample protocols have made the approach operationally tractable for the broad biomedical community [132]. Germline variants in IFNAR1, IFNAR2, OAS1, and IRF7 influence IFN-I signaling and innate antiviral responses, and the same loci have been implicated in differential outcomes of SARS-CoV-2 infection and vaccine response [133]. These variants provide genetic instruments for the vaccine-induced IFN-I cascade that drives antigen-agnostic effects on innate sensors [3]. Reporting under the STROBE-MR guidelines standardizes the documentation of instrument selection and assumption testing, including assessment of horizontal pleiotropy, weak-instrument bias, and the consistency of effect estimates across complementary analyses [134]. The combination of target trial emulation for the observational signal and Mendelian randomization for genetically anchored exposure effects constitutes a triangulation strategy capable of supporting causal claims that no single method could establish alone.

### 6.3. Pragmatic Trial Design for the Proposed Settings

**Figure 3:**
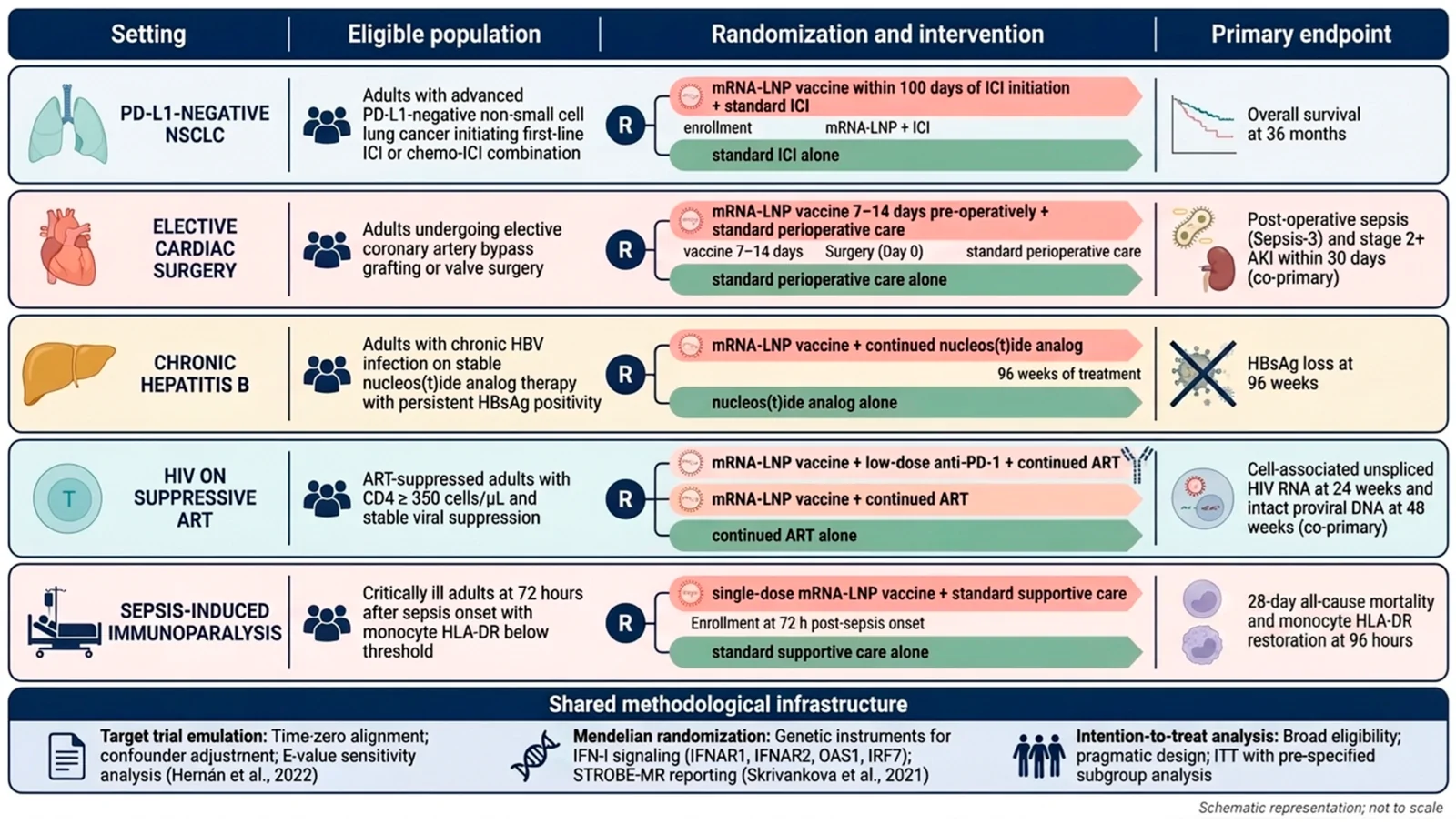
**Pragmatic trial design framework for the five proposed repurposing settings.** Operational architecture of pragmatic randomized trials designed to test the antigen-agnostic repurposing hypothesis across the five clinical contexts identified here. For each setting, the figure specifies the eligibility criteria, randomization scheme, intervention timing relative to standard-of-care therapy, and primary endpoint. In PD-L1-negative non-small cell lung cancer (NSCLC), randomization is to mRNA-LNP vaccination within 100 days of immune checkpoint inhibitor (ICI) initiation versus standard care, with overall survival as primary endpoint. In elective coronary artery bypass grafting (CABG) or valve surgery, randomization is to pre-operative mRNA-LNP administration 7 to 14 days before surgery versus no pre-operative vaccination, with post-operative sepsis and acute kidney injury (AKI) as co-primary endpoints. In chronic hepatitis B virus (HBV) infection on stable nucleos(t)ide analog therapy, randomization is to adjunctive mRNA-LNP vaccination versus nucleos(t)ide analog therapy alone, with hepatitis B surface antigen (HBsAg) loss at 96 weeks as primary endpoint. In antiretroviral therapy (ART)-suppressed adults with HIV, randomization is to mRNA-LNP vaccination with or without low-dose anti-PD-1 monoclonal antibody versus continued ART alone, with cell-associated unspliced HIV RNA at 24 weeks and intact proviral DNA at 48 weeks as co-primary endpoints. In critically ill adults with sepsis-induced immunoparalysis at 72 h after sepsis onset, randomization is to single-dose mRNA-LNP vaccination *versus* standard supportive care, with 28-day all-cause mortality and monocyte HLA-DR restoration at 96 h as co-primary endpoints. The shared methodological infrastructure across the five trials includes target trial emulation principles for time-zero alignment, confounder adjustment and E-value sensitivity analysis [128], Mendelian randomization for triangulation of causal estimates through genetic instruments for type I interferon (IFN-I) signaling with reporting under the STROBE-MR statement [134], and intention-to-treat (ITT) analysis with broad eligibility consistent with pragmatic design. Abbreviations: AKI, acute kidney injury; ART, antiretroviral therapy; CABG, coronary artery bypass grafting; HBsAg, hepatitis B surface antigen; HBV, hepatitis B virus; HLA-DR, human leukocyte antigen-DR; ICI, immune checkpoint inhibitor; IFN-I, type I interferon; ITT, intention-to-treat; LNP, lipid nanoparticle; mRNA, messenger RNA; NSCLC, non-small cell lung cancer; PD-1, programmed cell death protein 1; PD-L1, programmed cell death ligand 1. *Image generated with FigureLabs.ai Scientific Illustration Software (web-based application; accessed on August 2026).*

Pragmatic trial design adapts the randomized protocol to real-world clinical contexts, with minimal exclusion criteria, routine-care endpoints, and broad eligibility. Five settings warrant prospective evaluation (Table 2; Figure 3). In PD-L1-negative non-small cell lung cancer initiating first-line ICI, randomization to mRNA-LNP vaccination within 100 days of ICI initiation tests the antigen-agnostic adjuvant hypothesis with overall survival as primary endpoint [5]. In elective coronary artery bypass grafting or valve surgery, pre-operative administration 7 to 14 days before surgery tests the trained-immunity hypothesis with post-operative sepsis and acute kidney injury as co-primary endpoints [80]. In chronic hepatitis B virus infection on stable nucleos(t)ide analog therapy, adjunctive mRNA-LNP vaccination tests the TLR7/8 host-directed strategy with hepatitis B surface antigen loss at 96 weeks as primary endpoint [85]. In antiretroviral-suppressed adults with HIV and adequate CD4 reconstitution, mRNA-LNP vaccination with or without low-dose anti-PD-1 tests the exhaustion-reversal hypothesis with cell-associated unspliced HIV RNA and intact proviral DNA as co-primary endpoints [90]. In sepsis patients meeting biomarker-defined immunoparalysis criteria at 72 h after sepsis onset, a single dose of mRNA-LNP vaccine versus standard care tests the immune-restoration hypothesis with 28-day mortality and monocyte HLA-DR recovery as co-primary endpoints [99]. Each protocol is logistically tractable because the intervention is approved, manufactured at scale, and supported by extensive safety data [1].

### 6.4. Limitations of the Current Evidence Base

Several limitations of the current evidence base warrant explicit acknowledgment before prospective testing is initiated. First, all human efficacy signals to date derive from retrospective observational cohorts in which vaccination was neither randomized nor stratified by clinical risk. Patients who received a COVID-19 mRNA vaccine near ICI initiation likely differed systematically from unvaccinated patients in performance status, comorbidity burden, engagement with supportive care, and access to routine preventive services. This healthy-vaccinee bias is difficult to eliminate through covariate adjustment alone, and the residual confounding necessary to nullify the observed hazard reduction has not been formally quantified through E-value analysis in the published cohorts. The independent nationwide validation of 4407 patients confirmed the direction of effect [39] with an effect estimate that the authors interpreted as compatible with some degree of unmeasured confounding in the original signal. Second, the vaccine-induced PD-L1 upregulation invoked as the molecular substrate for ICI synergy has been documented in preclinical models and inferred from healthy volunteer immunology, although direct measurement of PD-L1 induction within a human tumor-draining lymph node after mRNA vaccination has not been reported. The anatomic and temporal alignment postulated for the model therefore remains an inference rather than a directly measured phenomenon. Third, the trained-immunity framework applied to the perioperative and sepsis contexts relies on cellular and transcriptional signatures that partially overlap with the BCG-induced phenotype. Durable epigenetic reprogramming of hematopoietic stem and progenitor cells of the kind described for BCG has not been demonstrated for mRNA-LNP vaccines in humans, and the duration of any trained-like reprogramming beyond the acute innate response window is undefined. Fourth, the extension of the antigen-agnostic hypothesis to chronic HBV rests on residual TLR7/8 signaling by nucleoside-modified mRNA. This signaling is present but attenuated relative to unmodified RNA, and neither the intensity nor the duration of TLR7 engagement is comparable to a purpose-designed, dose-titratable oral TLR7 agonist. The clinical proposal in that setting should therefore be regarded as a pharmacologic complement rather than an equivalent substitution. Similarly, the sepsis proposal invokes vaccine-induced IFN-I signaling as a mechanistic complement to the recombinant IFN-γ arm of the PROVIDE protocol, but IFN-I and IFN-γ engage distinct receptor systems and induce partially non-overlapping transcriptional programs. Pharmacodynamic monitoring of monocyte HLA-DR restoration is therefore essential to any prospective evaluation in this setting before mortality endpoints are trusted. Fifth, the perioperative cardiac surgery proposal requires careful attention to the temporal window between vaccination and surgical insult. Post-vaccination myocarditis typically presents 2 to 4 days after administration, which partially overlaps with the proposed 7 to 14-day pre-operative interval. Stratified dosing by age and sex, and pre-specified troponin thresholds for surgical postponement, would attenuate the risk of iatrogenic peri-operative myocardial injury. Finally, the methodological framework outlined above provides tools for causal inference but does not itself close the evidence gap. Target trial emulation improves the credibility of observational analyses without eliminating unmeasured confounding, and Mendelian randomization instruments for baseline IFN-I responsiveness rather than for vaccine-induced IFN-I induction specifically. The exclusion restriction assumption may be violated because IFNAR1, IFNAR2, OAS1, and IRF7 variants also influence infection susceptibility and severity independently of vaccine response. Randomized pragmatic trials therefore remain the definitive path to causal ascertainment.

## 7. Conclusions

Licensed COVID-19 mRNA-LNP vaccines should no longer be viewed solely as pathogen-specific prophylactic agents, but also as plausible host-directed immunomodulators whose biological effects may extend across distinct clinical contexts. The convergence of innate sensor engagement, IFN-I induction, dendritic-cell activation, and checkpoint-relevant immune remodeling provides a coherent mechanistic basis for this repositioning hypothesis, while the emerging oncologic signal suggests that these effects may already be clinically visible under the right temporal and biological conditions. At the same time, the inconsistency of retrospective findings makes clear that mechanistic plausibility is not itself sufficient evidence of therapeutic benefit.

The central implication of the available literature is therefore not that repurposing has been proven, but that it has become testable in a rigorous and immediately translatable way. Because the platform is already licensed, scalable, and supported by a safety database that few investigational agents can match, the decisive question is no longer feasibility, but clinical prioritization. Prospective evaluation should now focus on settings in which the immunobiology, unmet need, and trial logistics are most strongly aligned, particularly those in which transient reinforcement of innate or checkpoint-coupled immunity could plausibly shift clinically meaningful outcomes. In this sense, COVID-19 mRNA vaccines may represent an unusual repurposing candidate in which mechanistic rationale, operational readiness, and public-health scalability coincide, although none of these features substitutes for the prospective efficacy data that remain absent. What remains is to determine, through appropriately designed pragmatic trials and causal-inference frameworks, whether an intervention developed for antiviral prevention can be redeployed as a broadly useful therapeutic modulator of host immunity.

## Figures and Tables

**Table 1:** Clinical studies of COVID-19 mRNA vaccination in patients receiving immune checkpoint inhibitor therapy.

Study (Year, Country)	Design	Population (n; ICI-Treated; Cancer Types)	Vaccine	Primary Efficacy Finding	Principal Safety Finding
Grippin et al., 2025 (USA) [5]	Retrospective multi-cohort	n = 1166; advanced non-small cell lung cancer (NSCLC) and metastatic melanoma; analyses stratified by PD-L1 status	BNT162b2 or mRNA-1273 within 100 days of ICI initiation	Median OS extended in vaccinated patients across both cohorts (NSCLC and melanoma), with stronger effect in PD-L1-negative subgroup	No excess immune-related adverse events (irAE) versus unvaccinated control
Luo et al., 2025 (China) [57]	Retrospective single-center cohort	n = 394 NSCLC on PD-1/PD-L1 inhibitor	Predominantly inactivated (BBIBP-CorV, CoronaVac); only 22 of 394 patients received mRNA vaccine	No PFS gain at follow-up; OS not significantly different	Reduced incidence of immune-related arthritis and myocarditis in vaccinated group
Lundgren et al., 2025 (Sweden) [20]	Prospective cohort	n = 20 NSCLC patients on ICI (38 healthy controls); longitudinal immunogenicity	BNT162b2 primary series plus third dose	Third dose normalized humoral and cellular response to levels comparable to healthy controls	No vaccine-related serious adverse events
Obermannova et al., 2025 (multinational) [60]	Phase IV interventional (CoVigi trial)	n = 204; solid tumors on systemic anti-cancer therapy	BNT162b2 or mRNA-1273	T cell responsiveness preserved in patients on ICI, monoclonal antibody, tyrosine kinase inhibitor, and curative radiotherapy; chemotherapy emerged as principal pharmacologic suppressor of vaccine response	No excess severe adverse events across treatment subgroups
Nelli et al., 2025 (Italy) [63]	Prospective cohort (Vax-On-Third-Profile)	n = 429; solid tumors on anti-cancer therapy	BNT162b2 third dose	Venous thromboembolism (VTE) rates comparable to those of untreated patients overall; natural killer (NK) cell response pattern independently associated with thromboembolic events	NK pattern identifies stratification criterion rather than contraindication
Wang et al., 2024 (meta-analysis) [9]	Systematic review and meta-analysis (27 studies)	n = 8331 (4724 on ICI); mixed solid tumors	BNT162b2, mRNA-1273, ChAdOx1 (mixed)	irAE incidence 21.96% before versus 14.88% after vaccination; seroconversion rate 1.05-fold higher in ICI-treated than chemotherapy-treated patients (95% confidence interval 1.01–1.10; *p* = 0.02)	No signal of vaccine-induced amplification of immune toxicity
Chen X. et al., 2023 (China) [53]	Preclinical and translational	Tumor-bearing murine models and human samples	BNT162b2 analog with concurrent anti-PD-1 or anti-PD-L1	Anti-PD-1 or anti-PD-L1 restored antibody and cellular vaccine response to levels of tumor-free controls; effect mediated by recovery of follicular helper T cell frequency and germinal center activity	Mechanistic confirmation of bidirectional dialogue between ICI and mRNA vaccination
Fabbri et al., 2025 (Italy) [64]	Prospective observational (Vax-On-Third) with propensity-score matching	n = 226 (102 exposed *versus* 102 matched controls); metastatic NSCLC on first-line pembrolizumab or cemiplimab ± chemotherapy	BNT162b2 third or fourth dose or bivalent booster within 60 days before to 30 days after ICI	No overall PFS or OS difference (PFS HR 0.88, 95% CI 0.66–1.18; OS HR 0.81, 95% CI 0.59–1.09); OS benefit restricted to PD-L1 tumor proportion score ≥50% single-agent subgroup (HR 0.59, 95% CI 0.36–0.96, *p* = 0.034)	No difference in any-grade irAE between cohorts
Arbel et al., 2026 (Israel) [39]	Retrospective real-world cohort, Clalit Health Services	n = 4407; NSCLC and metastatic melanoma on ICI	BNT162b2 and mRNA-1273	Reproduced Grippin analytic framework [5]; confirmed OS association with peri-ICI mRNA vaccination in both tumor types in an independent nationwide dataset	Not specifically reported
Heudel et al., 2026 (France) [43]	Retrospective single-center cohort, Centre Léon Bérard	n = 281; advanced cancer on ICI in 2021 (47 vaccinated within 100 days pre-ICI)	BNT162b2 and mRNA-1273 within 100 days before ICI initiation	Median OS 51.0 versus 21.0 months (*p* = 0.012); adjusted HR 0.615 (95% CI 0.407–0.928, *p* = 0.021) on multivariable analysis; median follow-up 51 months	Not specifically reported
Dimitrov et al., 2026 (Bulgaria) [42]	Nationwide retrospective real-world cohort (Bulgarian Ministry of Health)	n = 1797; solid malignancies with prior COVID-19 hospitalization survivors; median follow-up 48.6 months	mRNA-based versus vector-based versus no vaccination	mRNA *versus* vector or no vaccine associated with lower long-term mortality (OR 0.41, 95% CI 0.28–0.61, *p* < 0.0001); effect concentrated in lung cancer + ICI (OR 0.45, 95% CI 0.20–0.98, *p* = 0.036)	Not specifically reported
Chen P.-H. et al., 2026 (Taiwan; multinational data) [40]	Retrospective target trial emulation, TriNetX Research Global Network	n = 7218 propensity-matched (3609 vaccinated versus 3609 unvaccinated); solid tumors on ICI	BNT162b2 or mRNA-1273 within 100 days before ICI	14% reduction in all-cause mortality (HR 0.86, 95% CI 0.80–0.93); 57% reduction in ICU admission (HR 0.43, 95% CI 0.25–0.73); E-value 1.74 for the primary immune-related adverse event outcome	22% increase in overall irAE (HR 1.22, 95% CI 1.17–1.28); 11% increase in severe irAE (HR 1.11); ocular irAE only organ system surviving false-discovery rate correction (HR 1.48, 95% CI 1.17–1.87)
Karabuga et al., 2026 (Turkey) [58]	Retrospective multicenter cohort	n = 138; metastatic driver-negative NSCLC on second-line nivolumab	Sinovac (virion-based) versus mRNA (BNT162b2 or mRNA-1273) versus both	No difference in objective response rate (*p* = 0.38), clinical benefit rate (*p* = 0.16), or PFS (*p* = 0.39) across vaccine platform groups	Grade 1–2 irAE more frequent among Sinovac recipients; grade 3–4 irAE comparable
Çelik et al., 2026 (Turkey) [41]	Retrospective single-center cohort	n = 88; recurrent stage IV NSCLC on second-line nivolumab	≥2 versus 0–1 mRNA vaccine doses within 6 months before ICI	PFS2 14.0 versus 9.6 months (*p* = 0.04); adjusted HR 0.52 (95% CI 0.31–0.88, *p* = 0.01)	Not specifically reported
Jee et al., 2026 (USA) [65]	Retrospective real-world cohort with landmark and sensitivity analyses, Memorial Sloan Kettering	n = 8368; solid tumors on ICI 2017–2022	mRNA vaccines with peri-ICI (100-day) window	Grippin framework [5] replicated survival association; association confined to 2021 and dissipated in 2022; benefit not specific to ICI (also observed with 5-fluorouracil and osimertinib); disappeared under 100-day landmark restricted to March 2021 onward; conclusion attributes original signal largely to pandemic-era selection bias	Not specifically reported

ICI, immune checkpoint inhibitor; ICU, intensive care unit; irAE, immune-related adverse event; NSCLC, non-small cell lung cancer; OR, odds ratio; OS, overall survival; PFS, progression-free survival; PD-1, programmed cell death protein 1; PD-L1, programmed cell death ligand 1; NK, natural killer; VTE, venous thromboembolism.

**Table 2:** Map of proposed repurposing settings for licensed COVID-19 mRNA lipid nanoparticle vaccines.

Setting	Target Population	Mechanistic Rationale	Pharmacologic Precedent	Primary Endpoint	Section
Cancer immunotherapy combination	Adults with advanced PD-L1-negative non-small cell lung cancer (NSCLC) initiating first-line ICI or chemo-ICI combination	Type I interferon (IFN-I)-driven dendritic cell activation, conventional type 1 dendritic cell (cDC1) cross-priming of CD8+ T cells against tumor antigens, and coordinated upregulation of programmed cell death ligand 1 (PD-L1) on tumor and stromal cells convert immunologically cold tumors into ICI-responsive substrate	mRNA cancer vaccines in melanoma [71] and glioblastoma [17]; retrospective oncologic signal [5]	Overall survival at 36 months	Section 3
Perioperative immune dysfunction in cardiac surgery	Adults undergoing elective coronary artery bypass grafting (CABG) or valve surgery	Pre-operative innate immune priming with trained-immunity reprogramming through monocyte and hematopoietic stem and progenitor cell epigenetic remodeling, attenuating surgical immunosuppression and post-operative infectious vulnerability	Bacillus Calmette-Guérin perioperative precedent [80,82]	Post-operative sepsis (Sepsis-3 criteria) and stage 2 or higher acute kidney injury within 30 days, co-primary endpoints	Section 4.1
Chronic hepatitis B host-directed strategy	Adults with chronic hepatitis B virus (HBV) infection on stable nucleos(t)ide analog therapy with persistent hepatitis B surface antigen (HBsAg) positivity	Toll-like receptor (TLR) 7 and TLR 8 stimulation by modified mRNA cargo induces IFN-α response sufficient to drive HBsAg clearance and immune-mediated hepatocyte clearance, in parallel with sustained nucleos(t)ide analog viral suppression	TLR7 agonist JNJ-64794964 phase 1 trial [85]; preclinical hepatotropic TLR7 agonist SA-5 [86]	HBsAg loss at 96 weeks	Section 4.2
HIV reservoir and exhaustion reversal	Antiretroviral therapy-suppressed adults with chronic HIV infection, CD4 count ≥ 350 cells/μL, and stable viral suppression	Vaccine-induced IFN-I cascade and TLR7/8 stimulation amplify host antiviral programs operative in the residual reservoir; innate reprogramming potentiates the modest latency reversal observed with immune checkpoint blockade alone	AIDS Malignancy Consortium 095 anti-PD-1 ± anti-CTLA-4 in 40 people living with HIV with concurrent advanced malignancies [90]; confirmatory simian immunodeficiency virus macaque dual blockade [92]	Cell-associated unspliced HIV RNA at 24 weeks and intact proviral DNA at 48 weeks, co-primary endpoints	Section 4.3
Sepsis-induced immunoparalysis	Critically ill adults at 72 h after sepsis onset meeting biomarker-defined immunoparalysis criteria (monocyte HLA-DR below threshold)	Vaccine-induced IFN-I cascade and trained-immunity reprogramming align with the host signaling deficit characteristic of late-phase sepsis, providing a low-cost off-the-shelf alternative to recombinant interferon-γ in biomarker-guided immune restoration	PROVIDE randomized trial of biomarker-guided personalized immunotherapy with anakinra or interferon-γ in sepsis [99]	28-day all-cause mortality and monocyte HLA-DR restoration at 96 h, co-primary endpoints	Section 4.4

cDC1, conventional type 1 dendritic cell; CABG, coronary artery bypass grafting; CTLA-4, cytotoxic T lymphocyte-associated protein 4; HBV, hepatitis B virus; HBsAg, hepatitis B surface antigen; ICI, immune checkpoint inhibitor; IFN-I, type I interferon; NSCLC, non-small cell lung cancer; PD-L1, programmed cell death ligand 1; TLR, Toll-like receptor.

## Data Availability

No new data were created or analyzed in this study. Data sharing is not applicable to this article.
